# Genomic contraction of the *LOX* gene family limits jasmonic acid biosynthesis and contributes to delayed flower bud opening in honeysuckle (*Lonicera japonica*)

**DOI:** 10.1093/hr/uhag078

**Published:** 2026-03-03

**Authors:** Zhenhua Liu, Conglian Liang, Congzhe Hou, Longfei Zhang, Jing Li, Luyao Huang, Gaixia Zhang, Shaobin Pan, Runzhu Li, Chao Liu, Yongqing Zhang, Jia Li, Gaobin Pu

**Affiliations:** Shandong University of Traditional Chinese Medicine, Shandong Province Jinan 250355, China; Shandong University of Traditional Chinese Medicine, Shandong Province Jinan 250355, China; Shandong University of Traditional Chinese Medicine, Shandong Province Jinan 250355, China; Shandong University of Traditional Chinese Medicine, Shandong Province Jinan 250355, China; Shandong University of Traditional Chinese Medicine, Shandong Province Jinan 250355, China; Shandong University of Traditional Chinese Medicine, Shandong Province Jinan 250355, China; Shandong University of Traditional Chinese Medicine, Shandong Province Jinan 250355, China; Shandong University of Traditional Chinese Medicine, Shandong Province Jinan 250355, China; Shandong University of Traditional Chinese Medicine, Shandong Province Jinan 250355, China; Shandong University of Traditional Chinese Medicine, Shandong Province Jinan 250355, China; Shandong University of Traditional Chinese Medicine, Shandong Province Jinan 250355, China; Shandong University of Traditional Chinese Medicine, Shandong Province Jinan 250355, China; Shandong University of Traditional Chinese Medicine, Shandong Province Jinan 250355, China

## Abstract

The delayed flower bud opening of *Lonicera japonica* ‘Huajin 6’ extends its harvest window and enhances agricultural value, yet the underlying molecular basis remains unclear. Here, we assembled a chromosome-level genome of ‘Huajin 6’ using PacBio sequencing and high-throughput chromosome conformation capture scaffolding (824.72 Mb, scaffold N50 = 91.2 Mb). Comparative genomic analyses revealed a subfamily-specific contraction of *lipoxygenase* (*LOX*) genes, particularly within the *9-LOX* clade, which is associated with a reduced jasmonate biosynthetic capacity during floral development. Transcriptomic and hormone profiling showed coordinated suppression of jasmonic acid (JA) biosynthesis-related genes and a marked reduction of JA and its bioactive derivatives during the transition from the complete white stage to flower opening. A JA-responsive co-expression module enriched in cell wall modification genes exhibited attenuated activation in ‘Huajin 6’. Functional assays further demonstrated that exogenous JA restored timely flower bud opening in both ‘Huajin 6’ and *L. macranthoides*, while heterologous expression of *Lonicera LOX* genes enhanced jasmonate accumulation in *Arabidopsis*. Together, these findings are consistent with a jasmonate threshold model in which *LOX* gene contraction constrains JA accumulation during floral transition, contributing to delayed flower bud opening and highlighting how genome structural variation influences hormone-dependent flowering dynamics.

## Introduction

Honeysuckle (*Lonicera japonica* Thunb.), also known as Japanese honeysuckle, is native to East Asia and widely cultivated in China, Japan, and Korea [1]. Owing to its twining growth habit, attractive bilabiate flowers, and extended flowering period, often last from May to September under field conditions, it has also been introduced as an ornamental species in regions, such as Brazil, Australia, and the United States [2]. In China, *L. japonica* has been used in traditional medicine for the treatment of exopathogenic wind-heat syndromes and infectious diseases [3]. Its flower buds are rich in bioactive metabolites, including chlorogenic acid, flavonoids, and iridoid glycosides, which exhibit antioxidant, antiviral, and anti-inflammatory activities [4–7].

The commercial and horticultural value of *L. japonica* is highly dependent on the developmental stage at harvest and display. While flower buds are preferred for medicinal use due to their high content of bioactive metabolites, including chlorogenic acid, flavonoids, and iridoid glycosides, prolonged bud stages and delayed flower opening also enhance ornamental performance by extending the flowering period and maintaining floral aesthetics. Notably, chlorogenic acid content peaks during the second white stage (SWS), whereas flower opening is accompanied by rapid degradation of key bioactive compounds [8–10]. Consequently, cultivars with delayed flower bud opening are desirable not only for extending the optimal harvest window in medicinal production but also for improving flowering longevity and display quality in horticultural and ornamental contexts. Traditional breeding programs have therefore selected for delayed-flowering phenotypes, exemplified by ‘Huajin 6’, which exhibits a prolonged complete white stage (CWS) and suppressed floral dehiscence [11, 12]. Despite its agronomic importance, the molecular basis underlying this delayed flower bud opening remains poorly understood, limiting opportunities for targeted genetic improvement.

Jasmonic acid (JA) is a central phytohormone regulating diverse developmental processes and stress responses, including root growth, reproductive development, and organ abscission [13–15]. In plants, JA biosynthesis is initiated by lipoxygenases (LOXs), which catalyze the oxygenation of alpha-linolenic acid (α-LA) to generate oxylipin precursors [14, 16, 17]. Accumulating evidence indicates that JA contributes to flower opening by promoting cell wall loosening and interacting with ethylene signaling pathways [14, 16, 17]. However, the role of JA in floral development is context dependent; for example, JA promotes flower opening in *Lilium* but delays senescence in *Dendrobium* [18, 19]. These observations suggest that JA-mediated regulation of floral traits is tightly controlled and species specific.

Recent chromosome-level genome assemblies of medicinal plants, including Chinese goldthread (*Coptis chinensis*) [20], opium poppy (*Papaver somniferum*) [21], and *Taxus chinensis* [22], have revealed lineage-specific whole-genome duplication (WGD) events that contribute to the expansion of secondary metabolite pathways. While these studies have advanced our understanding of metabolic evolution, how genome structural variation, including gene family expansion or contraction, interfaces with hormone biosynthesis to shape agronomic traits remains largely unexplored. In particular, the functional consequences of gene family contraction, a common but underappreciated evolutionary strategy, have received relatively little attention in perennial medical plants.

Delayed flower bud opening in *L. japonica* provides an attractive system for investigating the interplay between genome evolution and hormonal regulation. Transcriptomic studies in woody and ornamental species, such as *Pyrus pyrifolia*, *Prunus persica*, and *Rosa hybrida*, have implicated JA dynamic in bud dormancy release and floral development [23–25]. However, few studies have integrated comparative genomics, multi-omics analyses, and hormone profiling to elucidate the genetic and molecular basis of delayed flower opening in perennial medical species. Moreover, whether variation in JA biosynthesis gene family contributes to this trait remains unclear.

To address these questions, we generated a high-quality chromosome-level genome assembly of *L. japonica* ‘Huajin 6’ using PacBio long-read sequencing, high-throughput chromosome conformation capture (Hi-C), and full-length RNA-seq sequencing. This resource enabled systematic comparative analyses with published genomes of *L. japonica* ‘Sijihua’ and *L. japonica* ‘Lj10107428’ [26, 27], as well as its relative species *L. macranthoides* ‘Xianglei’ [28]. By integrating comparative genomics, transcriptomics, hormone metabolomics, and functional assays, we demonstrate that subfamily-specific contraction of the *LOX* gene family, particularly within the *9-LOX* clade, restricts jasmonate biosynthetic capacity during floral transition. This JA deficiency suppresses the activation of cell wall modification genes, thereby delaying flower bud opening and prolonging the harvest window. Our findings illustrate a genome-to-hormone regulatory mechanism underlying delayed flowering and provide a conceptual framework for precision breeding of medicinal plants through targeted manipulation of hormone biosynthetic pathways.

## Results

### Morphological characteristics of flower bud opening in *L. japonica* ‘Huajin 6’

**Figure 1:**
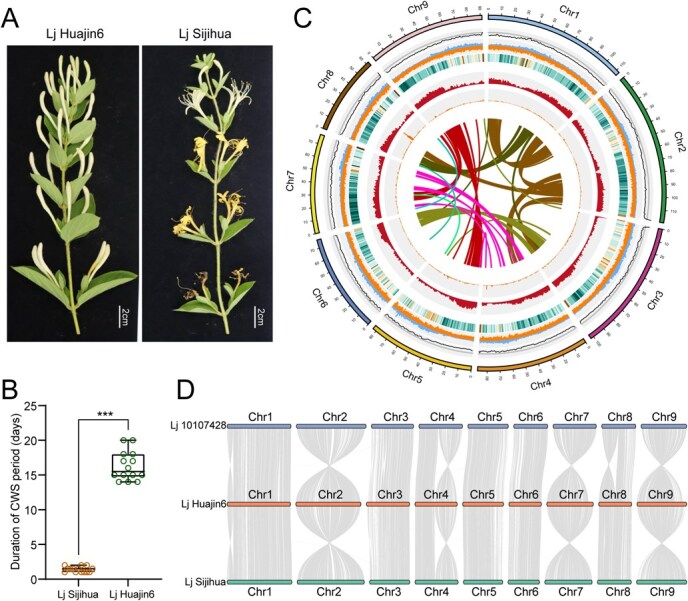
The flowering characteristics and genome assembly of *L. japonica* ‘Huajin 6’. (A) Macroscopic characteristics of fresh branches of *L. japonica* (Lj) Huajin 6 and Sijihua. (B) The duration of Lj Huajin 6 and Sijihua CWS. Each circle represents an individual; the data are presented as the mean ± SD (^***^P < 0.001, one-way ANOVA, *n* = 14). (C) Global view of the Lj Huajin 6. From outside to inside, the circles represent pseudochromosomes, GC contents, LTR-Gspsy density, LTR-Copia density, transposon element density, protein coding genes density, noncoding RNA genes density and syntonies connections. (D) Macrosynteny visualization between Lj Huajin 6 and Sijihua.

*Lonicera japonica* ‘Huajin 6’ is an elongated flower bud cultivar developed through long-term selective breeding (Fig. 1A). In contrast to *L. japonica* ‘Sijihua’, which exhibits a distinct silver flowering stage (SFS), ‘Huajin 6’ lacks a typical SFS and instead displays only a brief dehiscence stage (DS). Consequently, the CWS of ‘Huajin 6’ is markedly prolonged relative to ‘Sijihua’ (Fig. 1B). This extended CWS substantially increases the proportion of harvest flower buds, thereby enhancing harvesting efficiency and economic value.

### Chromosome-level genome assembly and annotation of *L. japonica* ‘Huajin 6’

A chromosome-level genome assembly of *L. japonica* ‘Huajin 6’ was generated by integrating PacBio long-read sequencing, Hi-C chromatin interaction data, and Illumina short-reads. K-mer analysis estimated a genome size of 854.54 Mb, with a heterozygosity of 1.02% and 42.66% high-copy repetitive sequences (Fig. S1, Table S1). In total, 119.90 Gb of Illumina reads (~140×), 92.08 Gb of PacBio reads (~108×), and 102.10 Gb of Hi-C data (~119×) were obtained, providing around 367× cumulative coverage of the estimated genome size (Tables S1–S3).

The final assembly produced using Falcon and Juicerbox consisted of 824.72 Mb, comprising 1143 contigs (contig N50 = 1.2 Mb) and 604 scaffolds (scaffold N50 = 91.2 Mb) (Table S4). Hi-C scaffolding anchored 98.46% (812.00 Mb) of the assembly onto 9 pseudochromosomes, with clear chromatin interaction patterns supporting assembly accuracy (Fig. S2, Table S5). BUSCO assessment identified 97.7% (1577/1614) of conserved embryophyta genes, indicating high completeness (Fig. S3).

Repeat annotation revealed that 491.86 Mb (60.57%) of the genome consists of repetitive elements, dominated by long terminal repeat retrotransposons (Gypsy, 15.03%; Copia, 13.49%) (Table S6, Fig. 1C). A total of 36 844 protein-coding genes were predicted, with an average transcript length of 4714 bp and a mean exon number of 4.71 (Table S7). Functional annotation was assigned to 94.34% of predicted genes based on homology and conserved domain analyses (Table S8).

Comparative genomics with previously published genomes of *L. japonica* ‘Sijihua’ [26] and *L. japonica* ‘Lj10107428’ [27] revealed extensive syntenic conservation (Fig. 1D). ‘Huajin 6’ shared 1247 syntenic blocks (41 338 collinear genes) with ‘Sijihua’ and 1189 syntenic blocks (41 693 collinear genes) with ‘Lj10107428’, confirming the structural reliability of the assembled genome.

### Genome evolution and gene family contraction in *L. japonica* ‘Huajin 6’

**Figure 2:**
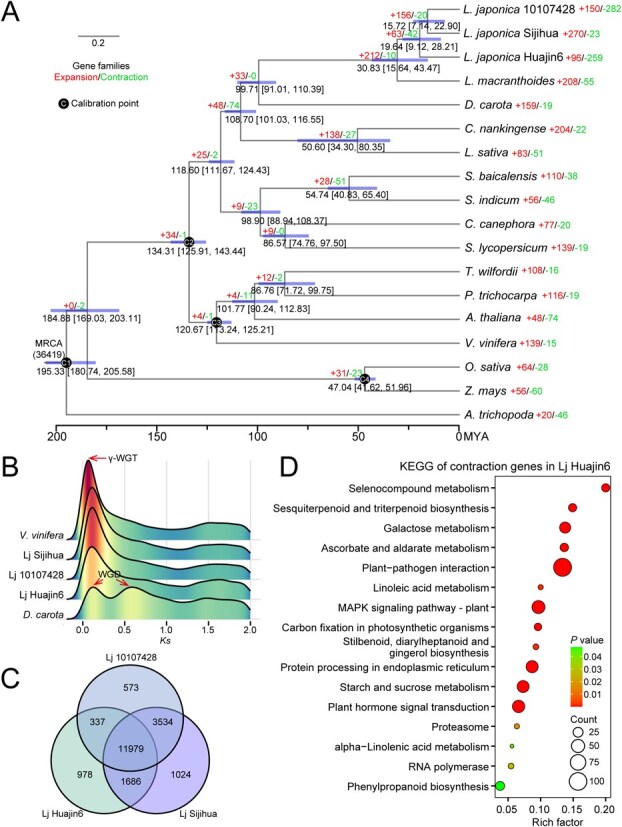
Phylogenetic analysis and whole genome duplication events of *L. japonica* Huajin 6*.* (A) Maximum-likelihood phylogenetic tree of Lj Huajing 6 and other 17 plant species, based on 263 shared single-copy genes. Divergence times were estimated based on four secondary calibration points (black circles) got from the TimeTree database. Horizontal bars denote confidence intervals. Gene family expansions and contractions are indicated by plus (+) and minus (-) signs preceding the numbers, respectively. (B) Ks distributions of paralogous genes within *L. japonica, D. carota*, and *V. vinifera.* (C) Venn diagram analysis of orthologous groups between Lj Huajin 6, Sijihua, and Lj10107428. (D) The KEGG pathways enrichment analysis of contracted genes in Lj Huajin 6. MYA, millions of years ago.

Orthologous gene clustering across *L. japonica* ‘Huajin 6’, ‘Sijihua’, ‘Lj10107428’, and 15 additional plant species identified 36 419 orthologous groups comprising 555 737 genes (Fig. S4, Tables S9 and S10). A phylogenomic tree constructed using 263 single-copy orthologs indicated ‘Huajin 6’ diverged from ‘Sijihua’ and ‘Lj10107428’ approximately 19.64 million years ago (Mya), while *L. japonica* and *L. macranthoides* diverged around 30.83 Mya (Fig. 2A). All three *L. japonica* cultivars shared conserved whole-genome triplication (γ-WGT) signatures, consistent with diploidized syntenic relationships relative to *Vitis vinifera* (Figs 2B and S5A and B).

Comparative analyses identified 11 979 conserved orthologous groups among the three *L. japonica* cultivars, with 978 groups unique to ‘Huajin 6’, including 6 enriched in secondary metabolite biosynthesis (Figs 2C and S6). Notably, 259 orthologous groups (787 genes) exhibited significant contraction in ‘Huajin 6’ (Fig. 2A). Kyoto Encyclopedia of Genes and Genomes (KEGG) pathway enrichment analysis of contrasted gene families revealed overrepresentation of pathways related to carbohydrate metabolism, plant–pathogen interaction, and phytohormone signaling (Fig. 2D). Importantly, genes involved in α-linolenic acid metabolism, an upstream pathway of jasmonate biosynthesis, were among the contracted groups.

### Subfamily-specific contraction of the *LOX* gene family

**Figure 3:**
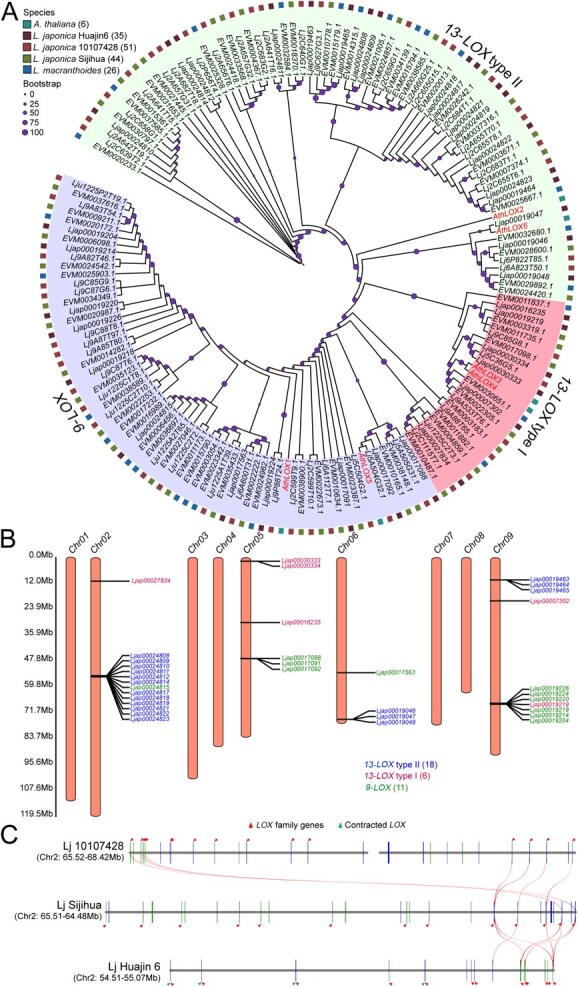
*LOX* family genes in *L. japonica.* (A) Phylogenetic tree of 162 *LOX* genes from *A. thaliana* (6), *L. japonica* ‘Huajin 6’ (35), *L. japonica* ‘Sijihua’ (44), *L. japonica* ‘Lj10107428’ (51) and *L. macranthoides* (26). (B) Chromosomal localization of *LOX* genes in Lj Huajin 6. (C) The collinearity relationship of *LOX* genes, between Lj Huajin 6, Sijihua, and Lj10107428. The blue and green lines represent genes in the forward and revise strands, respectively.

Given the enrichment of α-linolenic acid metabolism among contracted gene families, we further examined the *LOX* gene family. Using HMM-based identification (PF00305), we identified 35, 44, 51, and 26 *LOX* genes in *L. japonica* ‘Huajin 6’, ‘Sijihua’, ‘Lj10107428’, and *L. macranthoides* ‘Xianglei’, respectively. Phylogenetic analysis classified these genes into three clades corresponding to *Arabidopsis LOX* subfamilies: *9-LOX*, type I *13-LOX*, and type II *13-LOX* (Fig. 3A).

Quantitative comparisons revealed a pronounced reduction of *9-LOX* genes in ‘Huajin 6’ (11 copies) relative to ‘Sijihua’ (20 copies) and ‘Lj10107428’ (21 copies), whereas the numbers of type I and type II *13-LOX* genes remained relatively stable across cultivars (Fig. 3B). Chromosomal mapping showed that *LOX* genes in ‘Huajin 6’ are unevenly distributed, with enrichment on chromosomes 02, 05, 06, and 09. Notably, chromosome 02, syntenic to *LOX*-rich regions in other cultivars, lacked *9-LOX* genes entirely in ‘Huajin 6’ (Figs 3B and S7), suggesting localized gene loss events. Further comparative synteny analyses indicated a four-copy reduction in the type II *13-LOX* subfamily in ‘Huajin 6’ relative to ‘Lj10107428’ (Fig. 3C).

### Transcriptomic reprogramming associated with delayed flower bud opening

**Figure 4:**
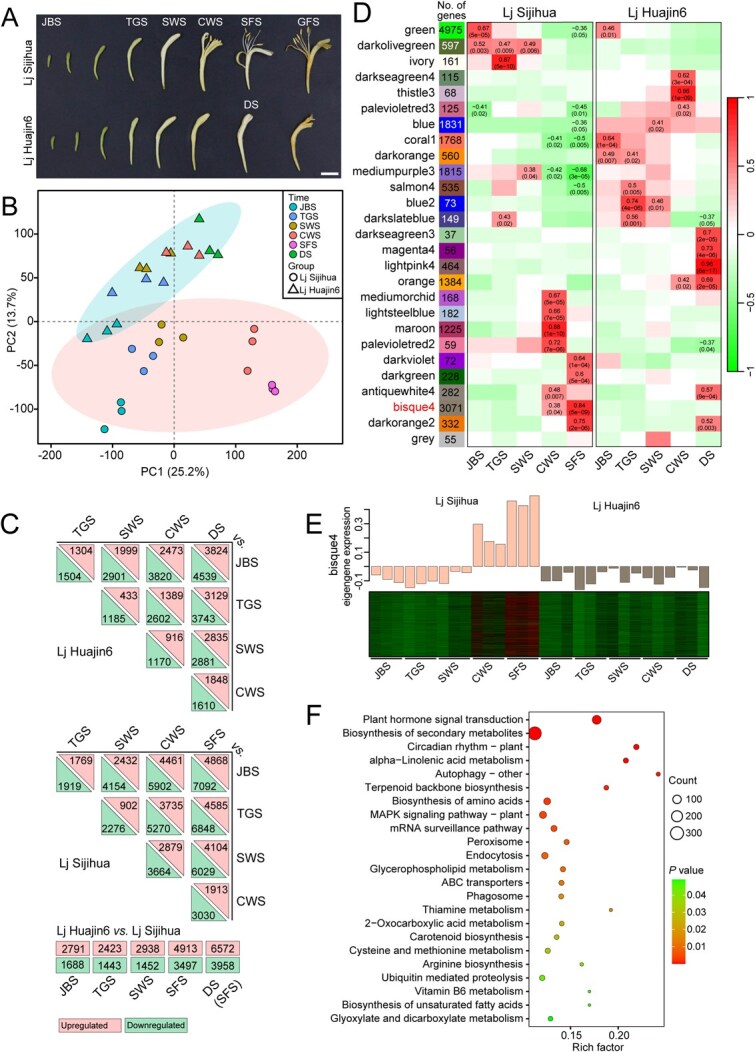
Transcriptome profiles of different flower bud development stages of *L. japonica* ‘Huajin 6’. (A) Macroscopic characteristics of flower buds of *L. japonica* ‘Huajin 6’ and ‘Sijihua’ in different stages. (B) PCA for all flower buds based on the expression values of transcriptome-wide expression profiles. (C) The number of DEGs over different stages. (D) Heatmap of module-development stages relationships analyzed by WGCNA. The right panel represents Pearson's correlation coefficient (see color scale). And the coefficients with the *P* value <0.05 were showed in the cells. (E) Eigengene expression profile for the ‘bisque4’ module in different stages. (F) KEGG pathways enrichment analysis of genes in ‘bisque4’ module.

To investigate transcriptional regulation underlying delayed flower bud opening, we profiled transcriptomes of ‘Huajin 6’ and ‘Sijihua’ across six developmental stages: juvenile bud stage (JBS), third green stage (TGS), SWS, CWS, SFS, and DS (Fig. 4A). Thirty libraries (three biological replicates per stage) were sequenced, generating 1.53 billion paired-end reads, with mapping rates exceeding 93% for all samples (Tables S11 and S12).

Principal component analysis revealed clear separation between developmental stages and between cultivars (Fig. 4B). Differential expression analysis identified 11 820 stage-specific differentially expressed genes (DEGs) in ‘Huajin 6’ and 17 178 in ‘Sijihua’, with transcriptional divergence increasing during later developmental stages (Fig. 4C). Weighted gene co-expression network analysis of 20 388 DEGs identified 26 modules (Fig. 4D). Among them, the bisque4 module (3071 genes) showed progressive activation during CWS and SFS in ‘Sijihua’ but remained transcriptionally inactive in ‘Huajin 6’ (Fig. 4E).

KEGG enrichment of bisque4 genes revealed significant overrepresentation of plant hormone signal transduction (*P* = 3.20 × 10^−9^), secondary metabolite biosynthesis (*P* = 1.70 × 10^−6^), and α-linolenic acid metabolism pathways (*P* = 4.80 × 10^−5^) (Fig. 4F). The strong enrichment for JA-related pathways, particularly in α-LA metabolism (JA precursor biosynthesis) and hormone signaling components, implies that dysregulation of JA biosynthesis and perception mechanisms may constitute a key regulatory node underlying differential bud opening kinetics between cultivars. These findings position phytohormonal regulation, especially JA-mediated signaling cascades, as critical determinants of flower bud opening progression in *L. japonica* ‘Huajin 6’.

### Reduced jasmonate accumulation during floral transition in ‘Huajin 6’

**Figure 5:**
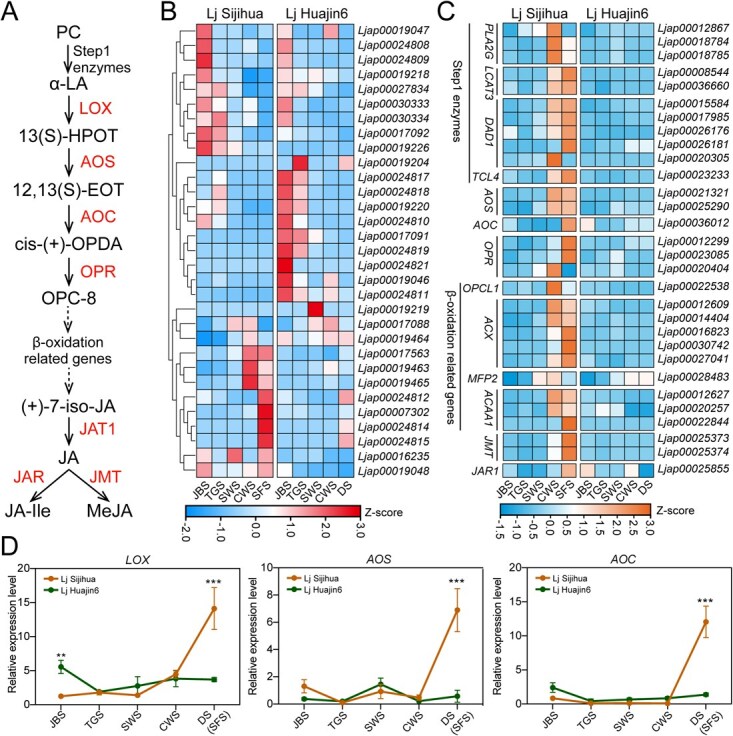
The biosynthetic pathway of jasmonic acid in *L. japonica* ‘Huajin 6’. (A) The JA biosynthetic pathway. (B–C) The expression level of all *LOX* family genes (B) and the key genes which high expressed in complete white and SFS (C) in different flower bud development stages for JA biosynthetic. (D) The expression level of the *LOX*, *AOC*, and *AOS* in different stages identified by RT-qPCR. Data are expressed as mean ± SD (^**^P < 0.01, ^***^P < 0.001, one-way ANOVA, n = 3).

Transcriptome analysis suggests that JA may play a role in regulating flower bud opening in *L. japonica* ‘Huajin 6’. We systematically analyzed the JA biosynthetic pathway to investigate this mechanism and compared differential gene expression patterns between *L. japonica* ‘Huajin 6’ and ‘Sijihua’ cultivars. The canonical JA biosynthesis pathway initiates with α-LA conversion through sequential enzymatic reactions involving LOX, allene oxide synthase (AOS), and allene oxide cyclase (AOC), ultimately producing 8-(3-oxo-2-(pent-2-enyl)cyclopentyl) octanoic acid (OPC8) which undergoes β-oxidation to form JA (Fig. 5A). Transcriptomic analysis revealed 35 *LOX* family genes in *L. japonica* ‘Huajin 6’, with 31 showing differential expression during bud development (Fig. 5B). Notably, 7 *LOX* genes exhibited elevated expression in critical developmental stages (CWS and SFS) of *L. japonica* ‘Sijihua’, coinciding with its active floral opening phase. Furthermore, we identified 30 JA biosynthesis-related genes from 13 enzyme classes showing preferential expression at developmental stages of CWS and SFS in *L. japonica* ‘Sijihua’, including 3 *AOS*, 1 *AOC*, and 3 *OPR* genes (Fig. 5C). Conversely, *L. japonica* ‘Huajin 6’ displayed distinct expression patterns, with 7 *dolichyl-diphosphooligosaccharide—protein glycosyltransferase subunit* (*DAD1*) and 2 *12-oxophytodienoic acid reductase* (*OPR*) genes showing peak expression during early developmental stages (JBS and TGS), followed by significant downregulation in later phases (Fig. S8).

**Figure 6:**
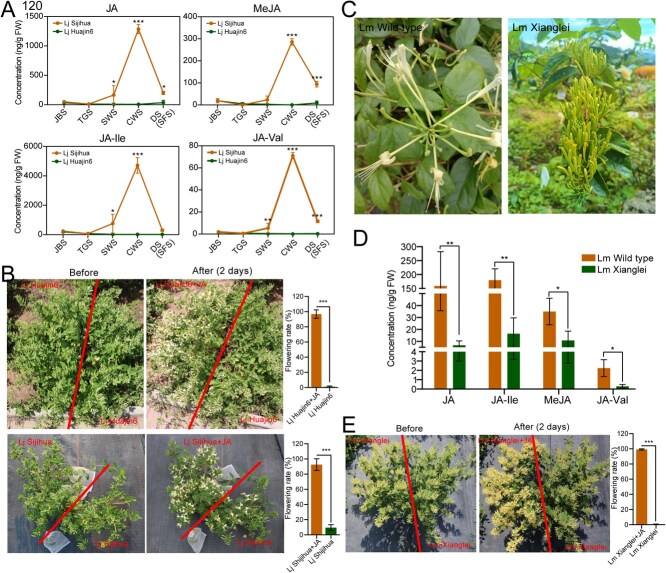
Jasmonic acid facilitates flower opening in *L. japonica and L. macranthoides.* (A) Concentration of JA, methyl jasmonate (MeJA), jasmonoyl-isoleucine (JA-Ile) and jasmonoyl valine (JA-Val) in different stages of *L. japonica* Huajin 6 and Sijihua flower buds. Data are expressed as mean ± SD (^*^P < 0.05, ^**^P < 0.01, ^***^P < 0.001, one-way ANOVA, *n* = 3). (B) Phenotypic comparison of *L. japonica* flowers before and after JA treatment. Bar graphs indicate flowering rate (%) after JA treatment. ^***^P < 0.001 (Kruskal–Wallis test, n = 5). (C) Macroscopic characteristics of flower buds of *L. macranthoides* (Lm) wild type and Xianglei. (D) Concentration of JA, MeJA, JA-Ile, and JA-Val in Lm wild type and Xianglei flowers. Data are expressed as mean ± SD (^*^P < 0.05, ^**^P < 0.01, one-way ANOVA, n = 3). (E) Phenotypic comparison of *L. macranthoides* flowers before and after JA treatment. Bar graphs indicate flowering rate (%) after JA treatment. ^***^P < 0.001 (Kruskal–Wallis test, n = 5).

The real-time quantitative PCR (RT-qPCR) validation confirmed comparable expression levels of *LOX*, *AOS*, and *AOC* between cultivars during early bud development. During later developmental stages, however, *L. japonica* ‘Huajin 6’ exhibited marked downregulation of these genes, with expression levels 3.8- to 5.2-fold lower than those in ‘Sijihua’ (Fig. 5D). Subsequent JA metabolite quantification revealed no significant inter-cultivar differences in JA, methyl JA (MeJA), JA-Isoleucine (JA-Ile), or JA-Valine (JA-Val) levels during early development (Fig. 6A). At the CWS stage, *L. japonica* ‘Huajin 6’ showed a dramatic reduction in JA derivative accumulation (JA, 4.1-fold; MeJA, 3.7-fold) compared to *L. japonica* ‘Sijihua’. In ‘Sijihua’, JA levels exhibited a transient peak during the CWS stage and returned to near-baseline levels at the SFS stage. Among the JA derivatives, JA-Val remained significantly elevated at SFS (2.9-fold higher than ‘Huajin 6’), whereas JA-Ile did not show sustained elevation at this stage. These results indicate that jasmonate accumulation during floral transition is transient and derivative-specific rather than persistently elevated.

Parallel phytohormone profiling demonstrated divergent patterns: salicylic acid (SA), gibberellin (GA) derivatives (GA53, GA20), and cytokinins (tZ, IP) decreased progressively in both cultivars, while abscisic acid (ABA) maintained relatively higher levels in *L. japonica* ‘Sijihua’ throughout development (Fig. S9A). Indole-3-acetic acid (IAA) exhibited a JA-like temporal pattern in ‘Sijihua’, peaking at the SWS stage. In leaves, no significant differences were observed in JA or its derivatives between ‘Huajin 6’ and ‘Sijihua’ (Fig. S10A), whereas ‘Huajin 6’ exhibited significantly reduced levels of SA and ABA (Fig. S10B).

### Exogenous JA application promotes flower bud opening

Functional validation through exogenous JA application (400 μmol/L) significantly accelerated floral opening in both cultivars compared to the control (Fig. 6B). Two days after treatment, nearly all flowers in the JA-treated group had opened, whereas those in the control group remained at the bud stage, representing a flowering rate of approximately 100% versus 0%. This promotive effect was specific to JA, as ABA or IAA treatments showed no such activity (Fig. S9C). Complementary observations in *L. macranthoides* ‘Xianglei’, a natural variant with delayed flowering (Fig. 6C), revealed analogous JA deficiency: JA derivatives were 58% to 67% lower than wild-type counterparts, while other hormones remained comparable except for reduced IP levels (Figs 6D and S9B). Exogenous JA restored expected flowering timing in *L. macranthoides* ‘Xianglei’ (Fig. 6E), confirming the conserved role of JA across *Lonicera* species.

### Heterologous expression of *LjLOX* genes enhances jasmonate accumulation in *Arabidopsis*

To evaluate the intrinsic biochemical competence of *Lonicera LOX* genes in promoting jasmonate biosynthesis, five representative *LjLOX* genes were selected for heterologous expression in *Arabidopsis thaliana*. These included members from all three major *LOX* subfamilies identified in *Lonicera*: *Ljap00024815* and *Ljap0001756*3 (*9-LOX*), *Ljap00007302* (type I *13-LOX*), and *Ljap00019463* and *Ljap00019465* (type II *13-LOX*), as determined by phylogenetic analysis. The selected genes were chosen to represent distinct *LOX* clades, to include members exhibiting differential expression during floral transition, and to incorporate *9-LOX* representatives reduced in copy number in ‘Huajin 6’.

Transgenic *Arabidopsis* lines expressing individual *LjLOX* genes accumulated significantly higher levels of endogenous JA and related jasmonates compared with empty-vector controls (Fig. S11). Although the magnitude of hormone increase varied among genes, all tested *LjLOX* genes enhanced jasmonate accumulation, indicating that diverse *LOX* subfamily members are functionally competent to promote JA biosynthesis.

## Discussion

Flower opening represents a tightly regulated developmental transition that integrates genome architecture with phytohormonal signaling [13–15, 29]. By combining chromosome-scale genome assembly, comparative genomics, transcriptomic profiling, hormone quantification, and functional assays, this study reveals that genome contraction, rather than expansion, can reprogram hormonal control of floral development. In *L. japonica* ‘Huajin 6’, a cultivar selected for delayed flower bud opening and an extended harvest window [11, 12], we identify a subfamily-specific contraction of *LOX* genes that coincides with a pronounced reduction in JA accumulation during the critical floral transition. These findings uncover an underappreciated evolutionary mechanism by which gene family loss quantitatively modulates hormone biosynthesis to shape developmental timing, complementing previous studies that emphasized gene family expansion in medicinal plants [20–22].

Among *LOX* family members, the preferential contraction of the *9-LOX* subfamily in ‘Huajin 6’ is particularly striking. This contraction is most likely attributable to localized loss of tandemly duplicated gene clusters rather than large-scale chromosomal rearrangements. As LOX enzymes catalyze the initial and rate-limiting step of oxylipin biosynthesis, they exert substantial control over the maximal capacity of jasmonate production [13, 14, 30]. From a gene dosage perspective, reduced *LOX* copy number may constrain the theoretical upper limit of JA biosynthetic flux during floral development. At the same time, transcriptomic analyses revealed stage-specific downregulation of multiple JA biosynthesis-related genes, including *LOX*, *AOS*, and *AOC*, in ‘Huajin 6’ during the critical floral transition, which likely further limits realized JA accumulation independent of copy number. Thus, genomic contraction may establish a constrained biosynthetic potential, whereas transcriptional regulation determines the stage-specific realization of hormone output. The reduced jasmonate levels observed in ‘Huajin 6’ are therefore best interpreted as the combined outcome of both gene dosage effects and transcriptional regulation, rather than being solely attributable to *LOX* copy number contraction. The absence of comparable JA deficiency in leaves further supports a tissue- and stage-specific limitation of JA biosynthesis rather than a systemic impairment of JA metabolism.

Importantly, the causal link between *LOX* gene contraction and JA deficiency is strengthened by functional evidence from heterologous expression analyses. Multiple *L. japonica LOX* genes, including those reduced or absent in ‘Huajin 6’, were individually expressed in *Arabidopsis* and consistently promoted the accumulation of endogenous JA and its bioactive derivatives. These results demonstrate that *Lonicera LOX* isoforms are intrinsically competent to drive JA biosynthesis, rather than merely being correlated with JA-associated transcriptional patterns. The convergent effects of distinct *LjLOX* genes further suggest functional redundancy within the *9-LOX* subfamily, supporting a gene-dosage-dependent model in which *LOX* copy number quantitatively determines JA biosynthetic flux. Similar gene-dosage effects of *LOX* family members on jasmonate output have been reported in *Nicotiana attenuata* and rice, where manipulation of *LOX* genes alters JA accumulation and downstream biological responses [31–36].

Jasmonates are known to participate in diverse aspects of plant growth, development and stress responses [13–19]; however, their role in regulating flower opening appears to be highly context dependent [18, 37]. Our data are consistent with a jasmonate threshold model, in which a transient increase in JA above a critical level may be required to activate downstream transcriptional programs associated with floral organ expansion. In ‘Sijihua’, JA accumulation peaks during the CWS and subsequently declines at the SFS, indicating that jasmonate signaling during flower opening is temporally restricted rather than persistently elevated. Although certain derivatives such as JA-Val remain relatively higher at SFS, JA-Ile does not exhibit sustained elevation at this stage. In ‘Huajin 6’, comparatively reduced jasmonate accumulation during the CWS is associated with attenuated activation of a JA-responsive co-expression module (bisque4) enriched in cell wall modification enzymes, including expansins and pectinases, thereby contributing to delayed bud opening. Although bisque4 is strongly associated with JA biosynthesis and signaling at the co-expression level, co-expression alone does not establish direct transcriptional regulation, and whether bisque4 genes represent primary JA-responsive targets or downstream components of JA-mediated developmental pathways remains to be determined. The observed temporal offset between peak JA accumulation and maximal expression of JA biosynthetic genes likely reflects the rapid and transient nature of jasmonate signaling, in which early JA accumulation can arise from pre-existing enzyme pools or post-translational activation of biosynthetic enzymes, whereas later transcriptional upregulation primarily reflects feedback regulation and sustained pathway flux rather than continued hormone accumulation [14, 18].

Although other phytohormones such as ABA, gibberellins, and cytokinins exhibited developmental dynamics, exogenous hormone application assays indicate that jasmonate availability represents the primary limiting factor for flower bud opening in this system. Hormonal crosstalk between JA and ABA or cytokinins has been documented in stress and developmental contexts [37–39], yet the complete restoration of timely flower opening by exogenous JA suggests that reduced jasmonate supply, rather than impaired signal integration, underlies the delayed flowering phenotype in *Lonicera*.

From an evolutionary and applied perspective, our findings illustrate how artificial selection can favor gene family contraction as an effective strategy to fine-tune developmental timing through hormone dosage control. In contrast to WGD, driven expansion of secondary metabolic pathways [20–22], the selective loss of *LOX* genes in ‘Huajin 6’ represents a distinct evolutionary trajectory that optimizes agronomic traits by attenuating jasmonate biosynthetic capacity. Together, these results support a model in which subfamily-specific *LOX* contraction quantitatively limits JA accumulation below the threshold required for flower bud opening, thereby prolonging the bud stage and extending the harvest window. This mechanism provides a conceptual framework for precision breeding strategies aimed at modulating flowering dynamics in ornamental and medicinal plants.

## Conclusion

This study reveals that delayed flower bud opening in *L. japonica* ‘Huajin 6’ is associated with subfamily-specific contraction of the *LOX* gene family, particularly the *9-LOX* clade, which constrains jasmonate biosynthetic capacity during floral development. Reduced JA accumulation at critical developmental transitions limits the activation of downstream gene networks required for flower opening, a mechanism supported by transcriptomic analyses, hormone profiling, and functional assays. Together, these results support a jasmonate threshold model in which genome-level modulation of hormone dosage governs the timing of flower bud opening. More broadly, our findings highlight gene family contraction as an effective evolutionary and breeding strategy for fine-tuning developmental traits and provide a conceptual framework for precision manipulation of flowering dynamics in ornamental and medicinal plants.

## Materials and methods

### Plant materials and sampling

Two *L. japonica* cultivars (‘Huajin 6’ and ‘Sijihua’) were collected from the Medicinal Botanical Garden of Shandong University of Traditional Chinese Medicine, Shandong Province, China. Fresh foliar tissue from healthy ‘Huajin 6’ plants were subjected to triple rinsing with ultrapure water to remove surface contaminants, immediately flash-frozen in liquid nitrogen, and stored at −80°C for genomic DNA extraction. Floral tissues from both cultivars were collected across six developmental stages: JBS, TGS, SWS, CWS, SFS, and DS. All samples were immediately flash-frozen in liquid nitrogen and stored at −80°C for subsequent analysis.

### Genome sequencing

Genomic DNA was extracted from the leaves of ‘Huajin 6’ using the Plant DNA Kit (Omega, USA). The integrity and concentration of the extracted DNA were evaluated by 0.8% agarose gel electrophoresis and quantified with a Qubit 4.0 Fluorometer (Thermo Fisher Scientific, USA), respectively. Genome survey libraries with insert sizes of 270 and 500 bp were constructed using the NEBNext Ultra II DNA Library Prep Kit (NEB, USA) and sequenced on an Illumina HiSeq X Ten platform in PE-150 mode, generating 172.40 Gb raw data. Following quality control with Falco v1.2.1 [40] and adapter trimming via Trimmomatic v0.38 [41], 119.90 Gb clean reads were retained for genome survey analysis.

A 40-kb SMRTbell library was prepared following PacBio’s standard protocol (Pacific Biosciences, USA) for long-reads sequencing. Sequencing on the PacBio Sequel IIe platform using five SMRT cells yielded 92.08 Gb of continuous long reads (CLRs) for genome assembly.

Hi-C libraries were constructed from cross-linked leaf tissue using the DpnII restriction enzyme (NEB, USA) following established protocols [42]. After Illumina HiSeq X Ten sequencing (PE-150 mode) and Trimmomatic trimming, 102.10 Gb clean reads were obtained for chromosomal scaffolding.

### Full-length transcriptome sequencing

Total RNA was extracted from various tissues of *L. japonica* ‘Huajin 6’, including flower buds, leaves, and stems, using the Plant RNA Kit (Omega, USA). Following quality assessment, equal amounts of total RNA from the three tissues were pooled for PacBio Iso-Seq library preparation using the SMRTbell Express Template Prep Kit 2.0 (Pacific Biosciences, USA). Sequencing on a single SMRT cell generated 18 447 825 circular consensus sequences (24.15 Gb).

### Genome assembly and quality assessment

The genome size was estimated through k-mer count distribution using the jellyfish v2.3.0 [43] and the GenomeScope v2.0 [44], next validated by the fingGSE (k-mer = 19) [45] under default parameter settings. Initial genome assembly was performed with the Falcon v0.7.0 [46] using PacBio CLR data, followed by three-step polishing: (i) PacBio consensus correction via the Arrow algorithm (GCpp v2.0.2), (ii) Illumina short-read polishing with the Pilon v1.22 [47], and (iii) redundancy removal using the Purge_haplotigs v1.1.1 [48]. Hi-C chromosomal scaffolding was conducted through the HiC-Pro v2.11.1 [49] data processing, the Juicer v1.6 [50] alignment, 3D-DNA v180922 [51] chromatin interaction analysis, and Juicebox v1.11.08 [52] manual curation, resulting in chromosome-level scaffolds. Assembly completeness was validated using BUSCO v5.0.0 [53] against the embryophyta_odb10 dataset composed of 1614 single-copy orthologs [54].

### Genome annotation

Repetitive elements were identified using EDTA v2.0.0 [55] and RepeatMasker v4.4.1 [56]. Gene prediction was performed through the integration of three approaches: (i) homology-based prediction using GeMoMa v1.6 [57] with protein sequences from *A. thaliana*, *Daucus carota*, *L. macranthoides* ‘Xianglei’, *L. japonica* ‘Sijihua’, and *L. japonica* ‘Lj10107428’; (ii) *ab initio* gene prediction using AUGUSTUS v3.2.2 [58]; and (iii) transcriptome-guided prediction employing GMAP v2018-03-25 [59] and PASA v2.3.3 [60]. Consensus gene models were generated using EVidenceModeler v24.0 [61], resulting in a high-confidence, non-redundant set of gene structure.

Functional annotation included the BLASTP v2.10.0 searches against the NCBI RefSeq non-redundant (NR) protein database, SwissProt, and KOG databases, supplemented by the InterProScan v5.52 [62]. Gene ontology (GO) terms were assigned via the Blast2GO v4.1.9 [63] and KEGG pathways through the KofamScan v1.3.0 [64].

### Comparative genomics and evolutionary analysis

Orthologous gene clusters among ‘Huajin 6’ and 17 representative plant species (Table S9) were systematically identified using OrthoFinder v2.5.4 [65], followed by the extraction of one-to-one orthologs (single-copy gene sets) for downstream analyses. Protein sequences of these conserved orthologous clusters were aligned using MAFFT v7.471 [66] with default parameters. To determine the optimal substitution model for phylogenetic reconstruction, protein sequence alignments were evaluated with ProTest v3.4.2 [67] under the Akaike information criterion (AIC), which selected the JTT + I + G + F model as the best-fit amino acid substitution matrix. A maximum-likelihood phylogenetic tree was subsequently constructed using RAxML v8.2.12 [68] based on concatenated alignments of 263 single-copy genes. Divergence times were estimated through Bayesian molecular dating with the MCMCTREE module in PAML v4.9j [69], incorporating fossil calibration points retrieved from the TimeTree database v5.0 [70]. Gene family expansions and contractions were analyzed using CAFÉ v4.2.1 [71], with a *P* value <0.05 considered indicative of statistically significant expansion or contraction. KEGG enrichment analysis of genes within significantly contracted families (*P* < 0.05) in the *L. japonica* ‘Huajin 6’ genome was performed using KOBAS v.3.0 (http://kobas.cbi.pku.edu.cn/).

### Whole-genome duplication analysis

To investigate paleopolyploidization events, synteny analyses were conducted between the ‘Huajin 6’ genome and two reference genomes: grapevine (*V. vinifera*), which lacks post-γ WGD events [72], and carrot (*D. carota*), which underwent two pre-γ WGDs [73]. Putative orthologs were initially identified using BLASTP (e-value ≤1e-5; top 10 hits) and subsequently filtered through MCScanX v1.0.0 [74] to detect collinear blocks. Synonymous substitution rates (Ks) for syntenic gene pairs were calculated using the ParaAT v2.0 [75] for sequence alignment and KaKs_Calculator v2.0 [76] with default parameters for evolutionary rate estimation. Chromosomal collinearity relationships were visualized using the JCVI v1.0.5 toolkit [77], enabling comparative genomic architecture assessment.

### *LOX* family genes identification

The HMM PF00305 for LOX was retrieved from the Pfam database (http://pfam.janelia.org/). HMMsearch (HMMER v3.3.12) [78] identified LOX homologs in the protein sequences of *L. japonica* ‘Huajin 6’, ‘Sijihua’, ‘Lj10107428’, *L. macranthoides* ‘Xianglei’, and *A. thaliana,* applying an e-value threshold of 1e-10*.* Multiple sequence alignment of LOX proteins was performed using the FastTree v2.1 [79] for phylogenetic tree construction, with visualization conducted through the iTOL toolkit v1.1.9 [80]. Genomic localization of *LOX* genes was mapped using the TBtools v2.142 [81].

### Transcriptome profiling of *L. japonica* ‘Huajin 6’ and ‘Sijihua’ flower buds

Total RNA was isolated from flower buds at various developmental stages using the Plant RNA Kit (Omega, USA). Thirty RNA libraries with an average insert size of approximately 350 bp were constructed using the TruSeq RNA Sample Preparation Kit v2 (Illumina, USA) and sequenced on an Illumina NovaSeq 6000 platform in PE-150 mode. After quality control with Falco v1.2.1 and adapter trimming using Trimmomatic v0.38, 228.83 Gb clean reads were retained for genome mapping. HISAT2 v2.2.1 [82] aligned these reads to the ‘Huajin 6’ genome assembly, followed by gene expression quantification using featureCounts v2.0.3 [83]. DEGs were identified with DESeq2 v1.28.1 [84], applying thresholds of adjusted *P* ≤ 0.05 and |log2(fold change)| ≥ 1 to define statistical significance.

WGCNA v1.73 [85] was implemented with parameters: softPower = 10, minModuleSize = 30, deepSplit = 2, and MEDissThres = 0.25 to perform gene co-expression network analysis. This classified 20 387 differentially expressed genes (DEGs) into 27 modules (including the grey unclassified module). Module-trait correlations were analyzed using module eigengenes (MEs) representing principal components of expression profiles. GO terms and KEGG pathway enrichment for module genes were conducted through the TBtools v2.142.

### Real-time quantitative PCR

Reverse transcription was carried out on 1 μg of total RNA using the Reverse Transcription Kit (Promega, USA) at 60°C for 35 minutes, according to the manufacturer’s protocol. Quantitative PCR (qPCR) analysis was performed using SYBR Green Dye (Takara, China) on a StepOne Real-time PCR system (Applied Biosystems, USA). Primer sequences for *LOX*, *AOC*, and *AOS* are listed in Table S13. Gene expression levels were normalized to *Actin* and quantified using the 2^−ΔΔCt^ method [86].

### Plant hormone quantification

Fresh leaves or flower buds from three independent biological replicates were ground into powder in liquid nitrogen. Approximately 50 mg of the powder was extracted by adding 1 mL of methanol/water/formic acid (15:4:1, v/v/v). The resulting extract was dissolved in 100 μL of 80% methanol, filtered through a 0.22-μm membrane filter, and analyzed by Metware Biotechnology (Metware, China) using an ExionLC™ AD UPLC system coupled to an Applied Biosystems 6500 Q TRAP mass spectrometer. Chromatographic separation was carried out on an Agilent SB-C18 column (2.1 × 100 mm, 3 μm) maintained at 40°C, with 2 μl injection volume. The mobile phase consisted of 0.1% formic acid in water (solvent A) and 0.1% formic acid in acetonitrile (solvent B), delivered at a flow rate of 0.35 ml/min. The gradient elution program was as follows: 5% A/95% B was maintained from 0 to 9 minutes; this ratio was held constant from 9 to 10 minutes; it was then linearly changed to 95% A/5% B from 10 to 11.1 minutes and remained until 14 minutes for complete elution. Mass spectrometry detection was performed in dynamic multiple reaction monitoring mode, and the online coupling of the chromatographic effluent to the mass spectrometry system was achieved via the electrospray ionization (ESI) source.

The data were acquired using Analyst v1.6.3 (AB SCIEX, USA). Then, the peak areas of different samples were quantified by Multiquant v3.0.3 (AB SCIEX, USA), and the peak areas were brought into the standard curve linear equation to calculate the phytohormone content.

### JA treatment

The plants of ‘Huajin 6’ and ‘Sijihua’ at the JBS stage were, respectively, foliar-sprayed with 400 μmol/L JA [11], 100 μmol/L ABA, 50 μmol/L IAA, or 10 μmol/L IP solutions. The control plants received equivalent applications of water. The flowering progression was monitored for 48 hours post-treatment. During exogenous spraying of plant hormone solutions, adjacent treatment areas were physically separated by plastic partitions to prevent cross-contamination between different experimental groups. Concurrently, the ground surface was covered with plastic sheeting to prevent leaching of hormone solutions into the soil, thereby eliminating environmental contamination.

### Heterologous expression of *Lonicera LOX* genes in *Arabidopsis*

Heterologous expression of *Lonicera LOX* genes in *A. thaliana* was performed essentially as previously described [87], with minor modifications. In brief, the coding sequences of *Ljap00024815* (336 bp), *Ljap00019463* (324 bp), *Ljap00019465* (1740 bp), *Ljap00007302* (2385 bp), and *Ljap00017563* (2664 bp) were individually cloned downstream of the CaMV 35S promoter in the binary vector pGFPGUSplus [88]. The resulting constructs were introduced into *A. thaliana* ecotype Col-0 via *Agrobacterium tumefaciens*–mediated floral dip transformation [89]. Transgenic plants carrying the empty pGFPGUSplus vector were generated in parallel and used as negative controls. For jasmonate quantification, whole rosette leaves from 3-week-old transgenic *Arabidopsis* plants were harvested and subjected to hormone analysis. At this stage, no obvious alterations in flowering time, flower opening, or floral morphology were observed in the heterologous overexpression lines compared with empty-vector controls.

### Statistical analyses

Data are presented as mean ± standard deviation of at least three replicates. Statistical analyses were conducted using SPSS 22.0 (SPSS Inc., USA), applying either the Kruskal-Wallis test or one-way ANOVA, with statistical significance set at *P* ≤ 0.05.

## Supplementary Material

Web_Material_uhag078

## Data Availability

All the relevant data supporting the findings of this study are available in the paper and supplementary data. The sequence data in this study can be found in the National Genomics Data Center under accession number PRJCA041333.
