# Supplementary material for: Genomic contraction of the LOX gene family limits jasmonic acid biosynthesis and contributes to delayed flower bud opening in honeysuckle (Lonicera japonica)
Source: Hortic Res. 2026 Mar 3;13(6):uhag078. doi: 10.1093/hr/uhag078 (PMC13254203; doi:10.1093/hr/uhag078)
Supplement: Web_Material_uhag078 [file web_material_uhag078.zip › Supplementary_Figures.docx]

**Supplementary files**

**
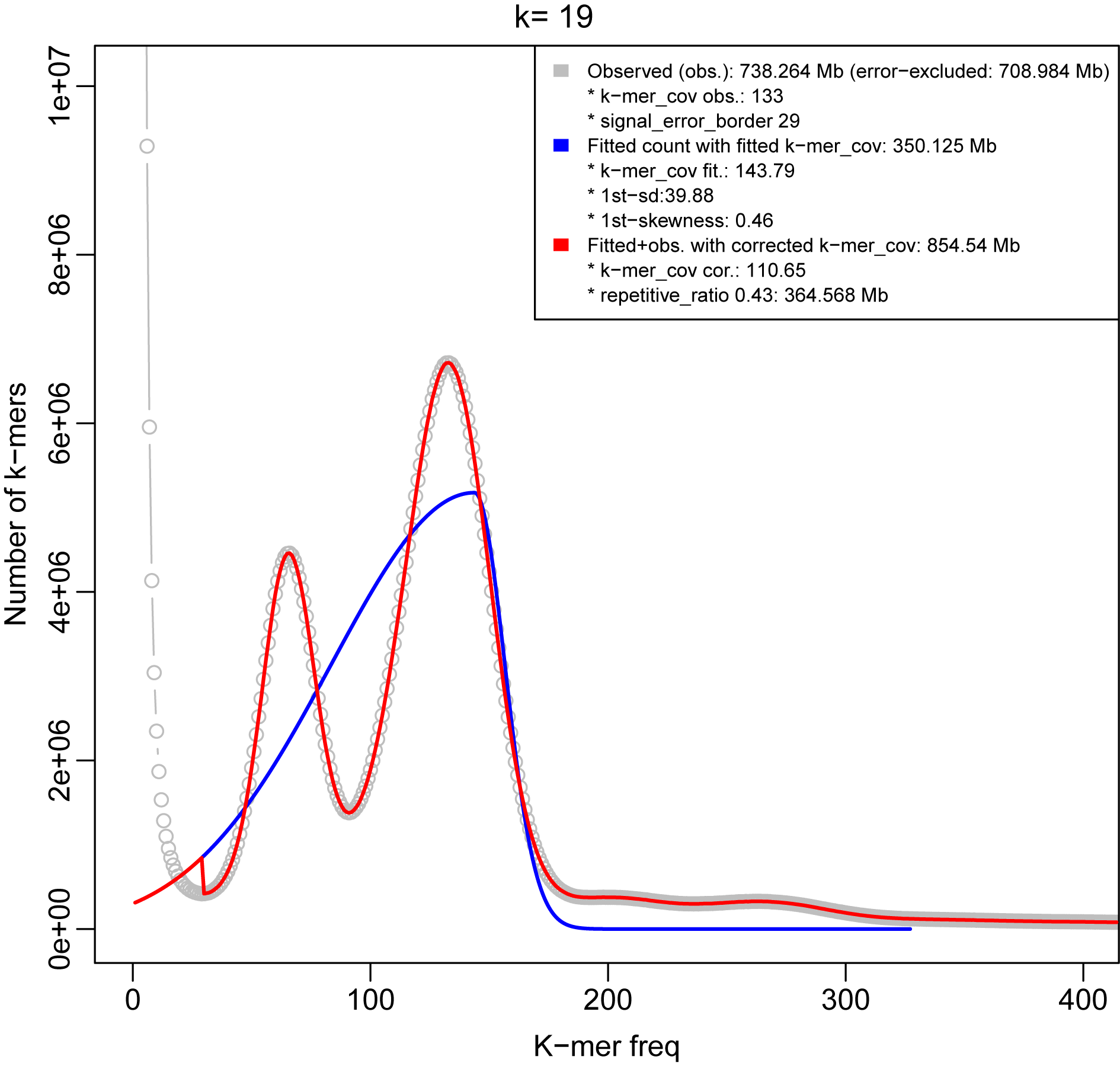
Figure S1**. Evaluation of the genome size of *Lonicera japonica* ‘Huajin 6’ by the Jellyfish and findGSE.

**
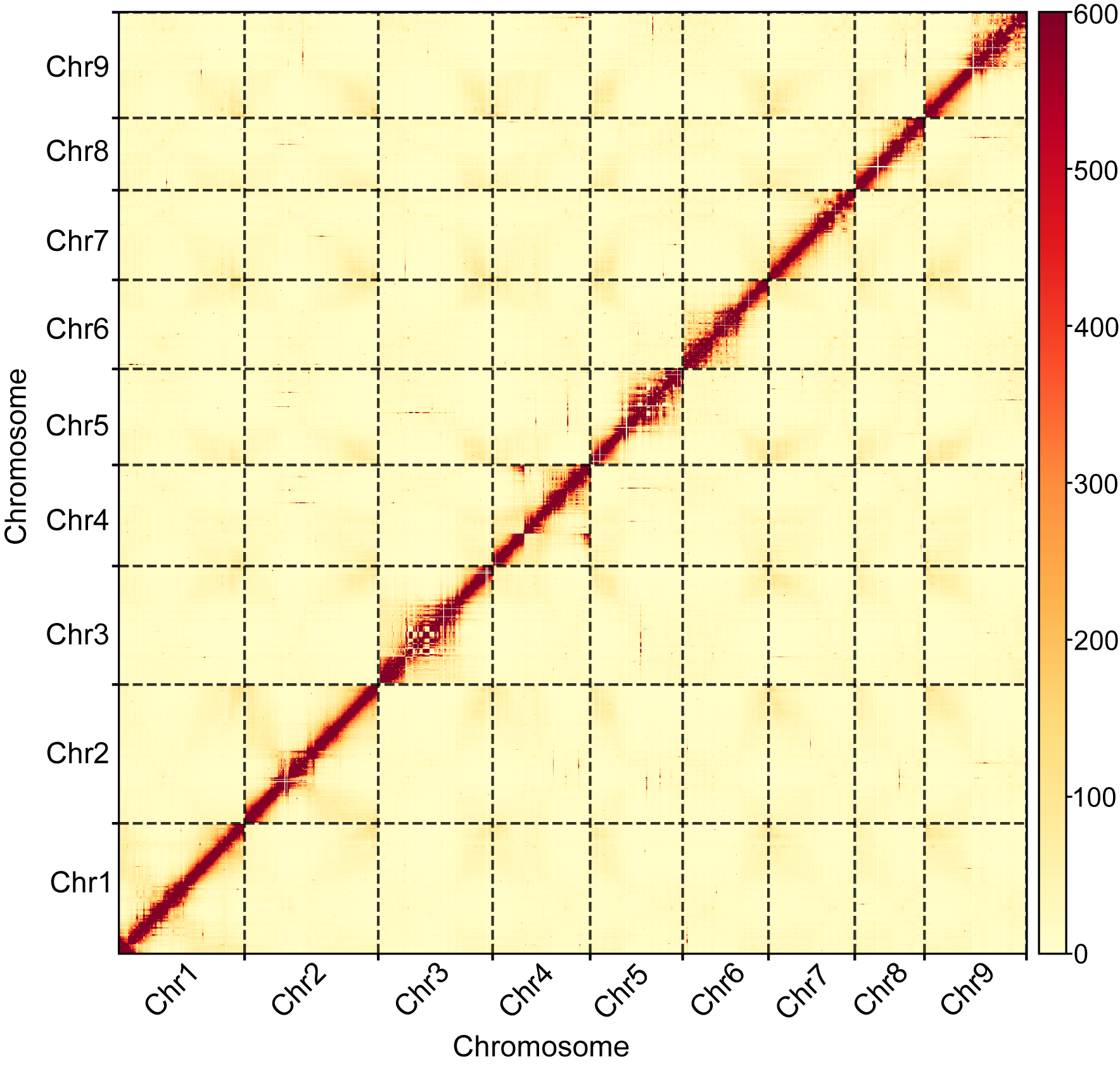
**

**Figure S2**. Hi-C intra-chromosomal contact map of the *Lonicera japonica* ‘Huajin 6’ genome assembly.


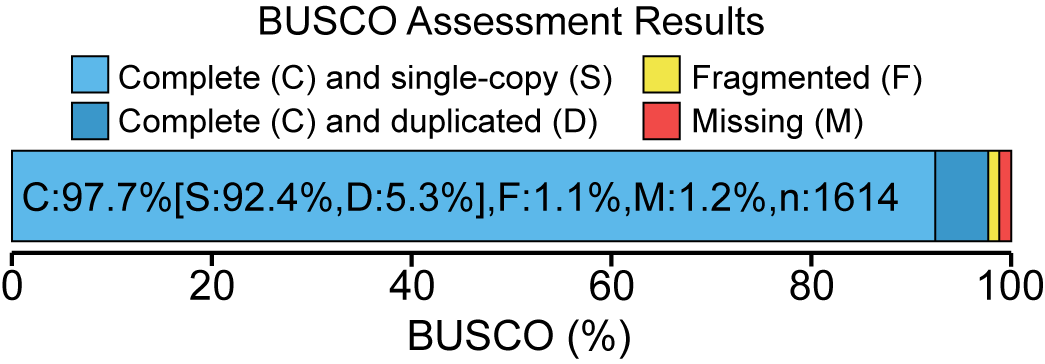


**Figure S3.** The quality estimation of genome assembly for *Lonicera japonica* ‘Huajin 6’ using BUSCO database.

**
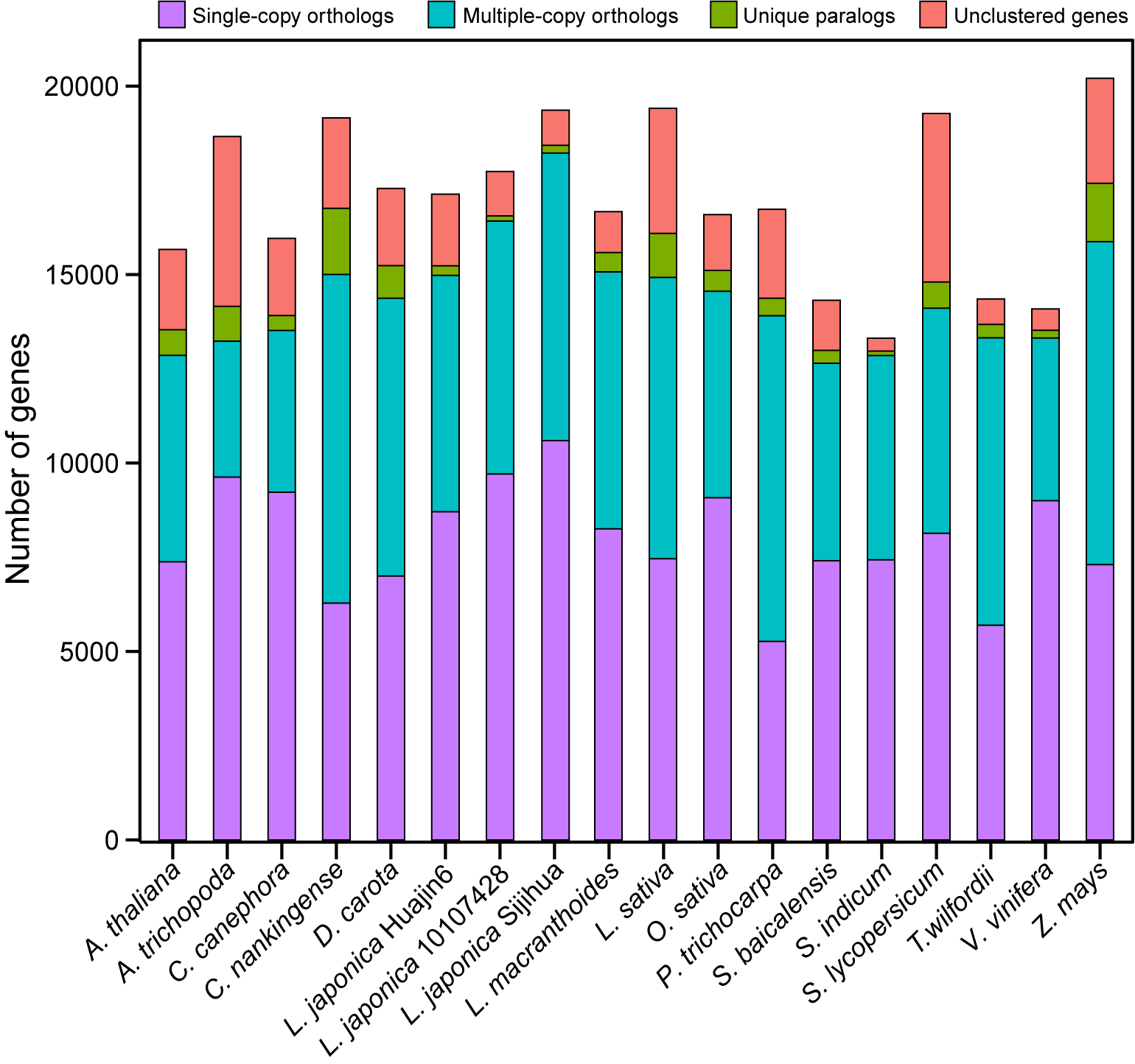
**

**Figure S4.** Clusters of orthologous gene families in *Lonicera japonica* ‘Huajin 6’ and other 17 plant species.


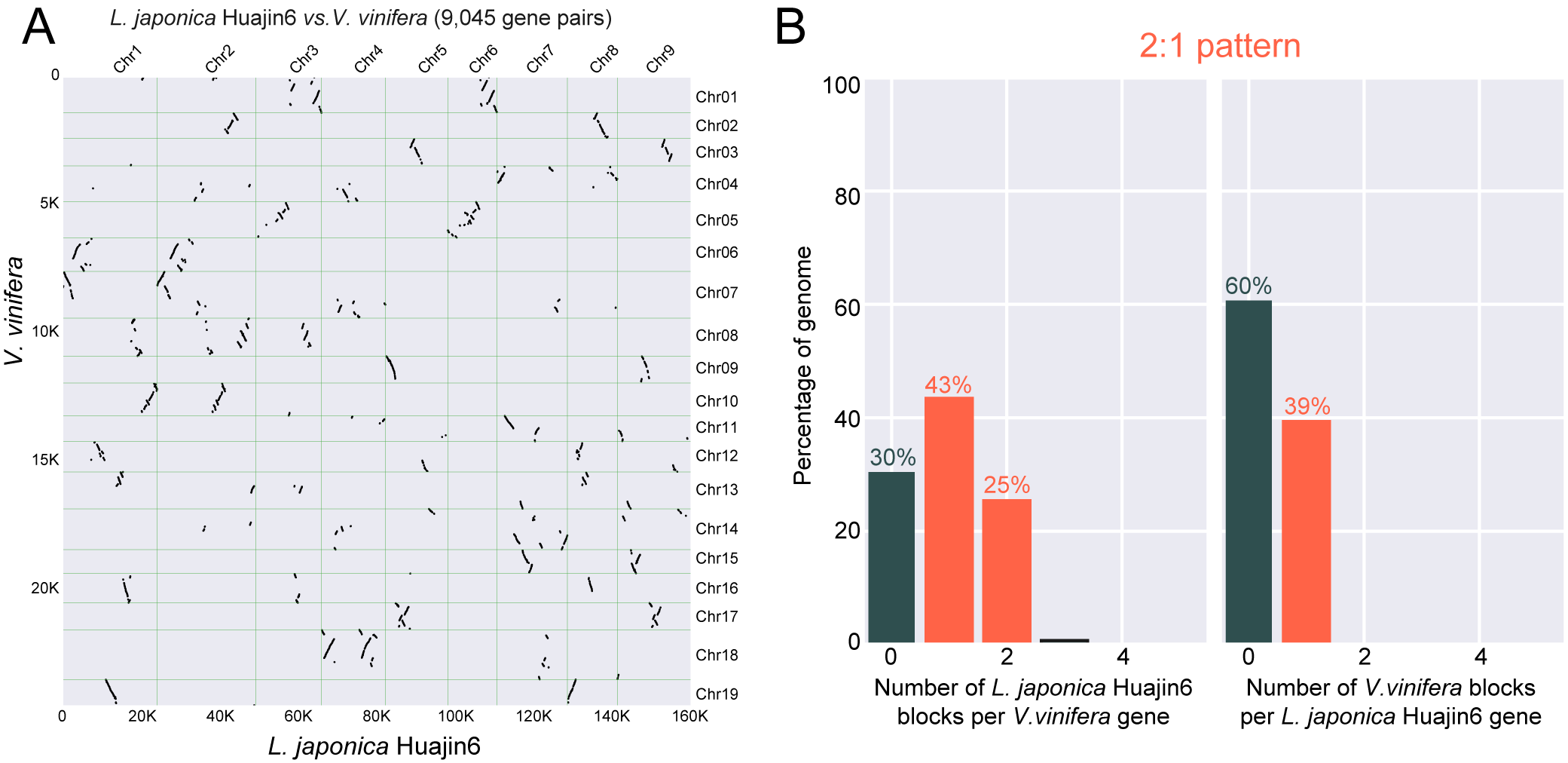


**Figure S5.** Syntenic analysis between *Lonicera japonica* ‘Huajin 6’ and *Vitis vinifera*. (A) The interspecies synthetic depths between two genomes. (B) The dot plot showing the syntenic regions in the inter-genomic comparison of two genomes.


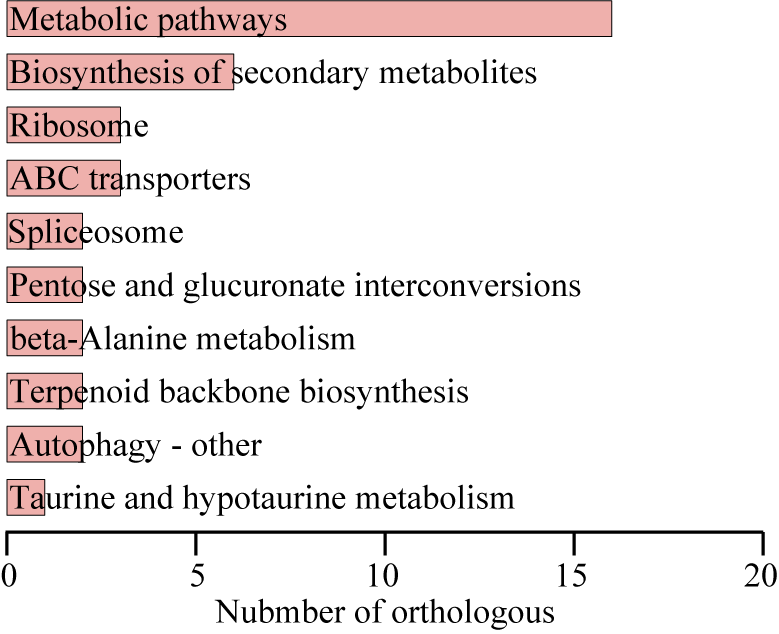


**Figure S6**. The Kyoto Encyclopedia of Genes and Genomes (KEGG) pathways enrichment analysis of 978 unique orthologous in Lj Huajin 6.


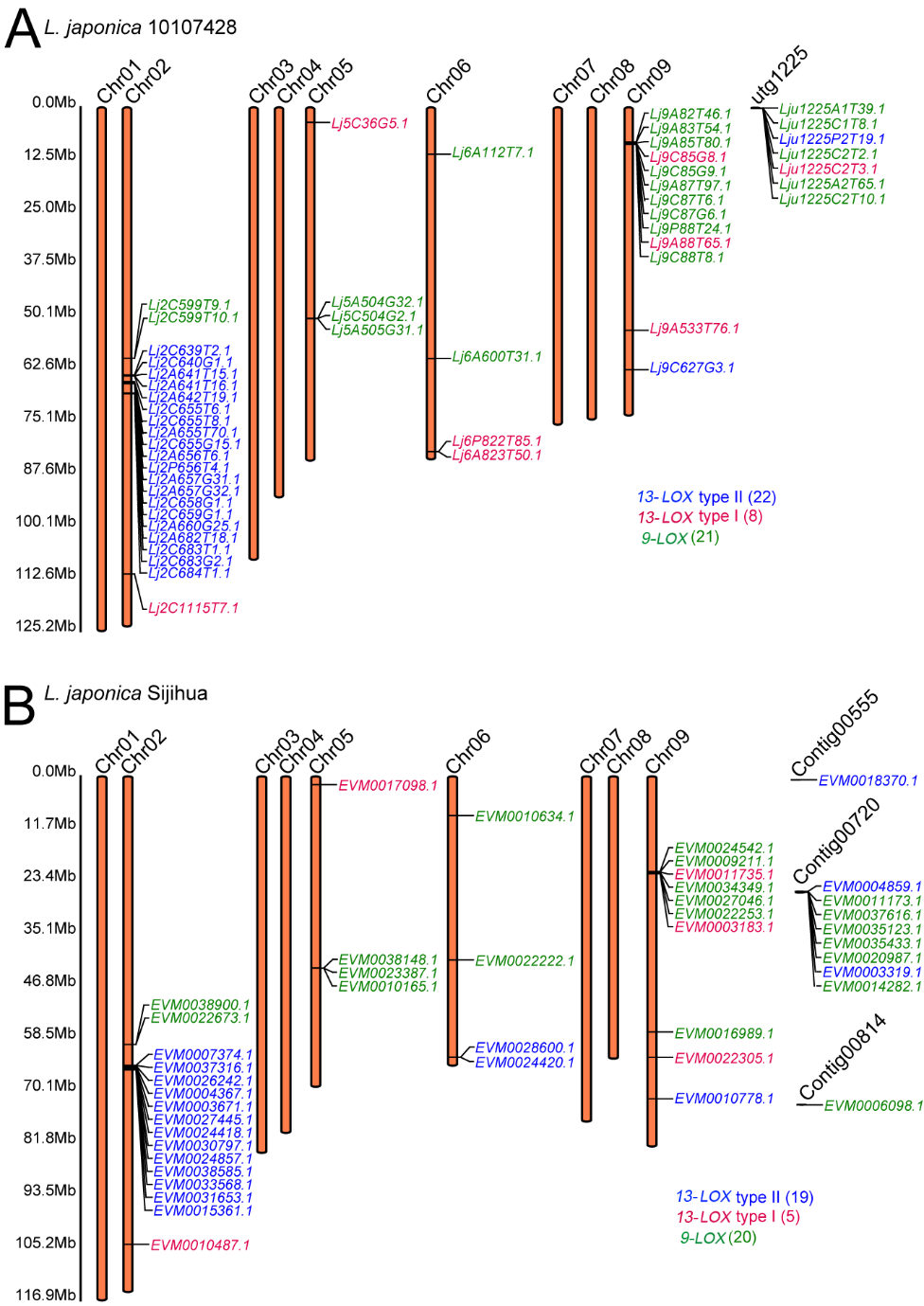


**Figure S7.** Chromosomal localization of *LOX* genes in *Lonicera japonica* ‘Lj10107428’ (C) and *L. japonica* ‘Sijihua’ (B).


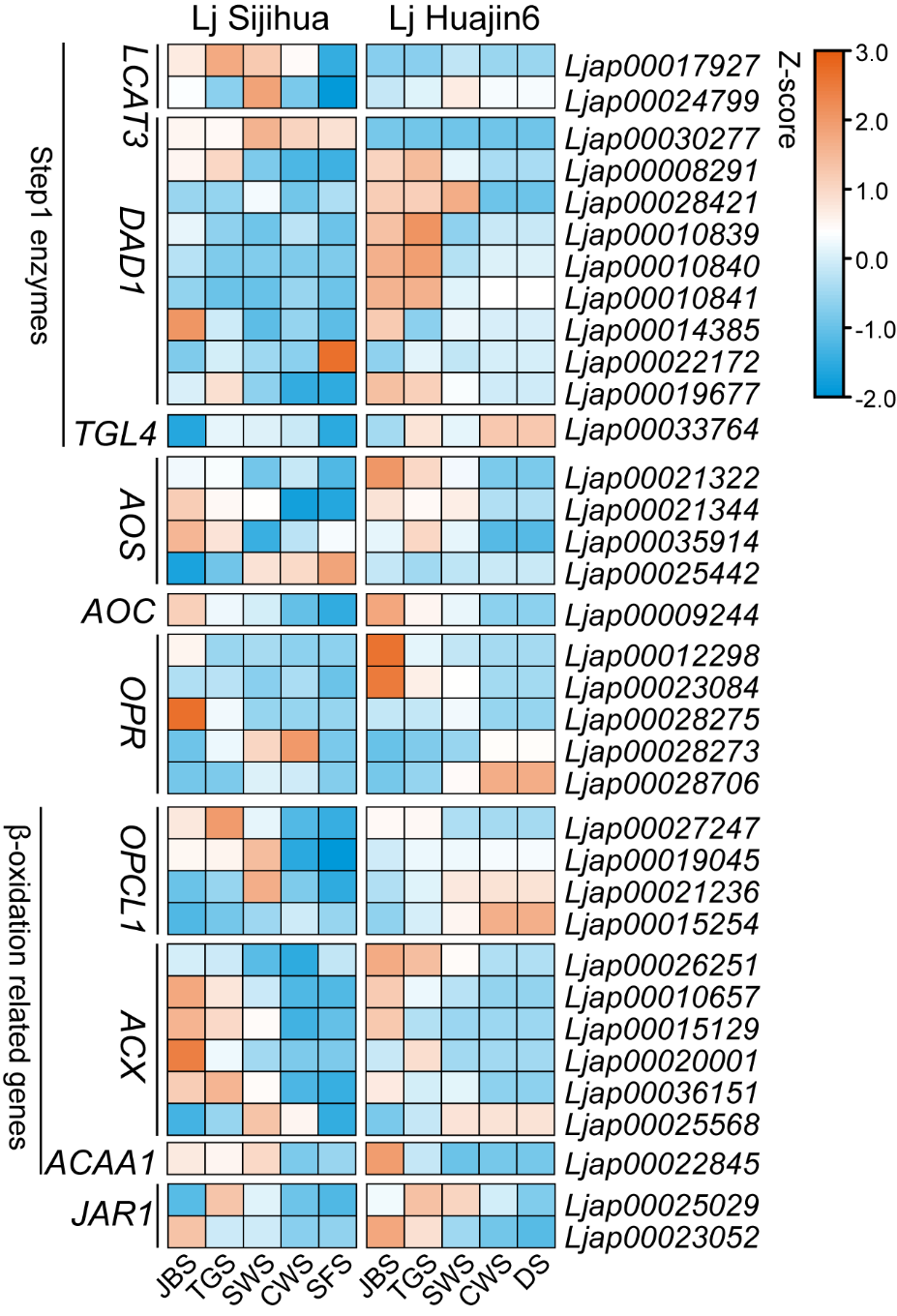


**Figure S8**. The key genes that are not highly expressed in complete white and silver flowering stages in different flower bud development stages for jasmonic acid biosynthetic.

**
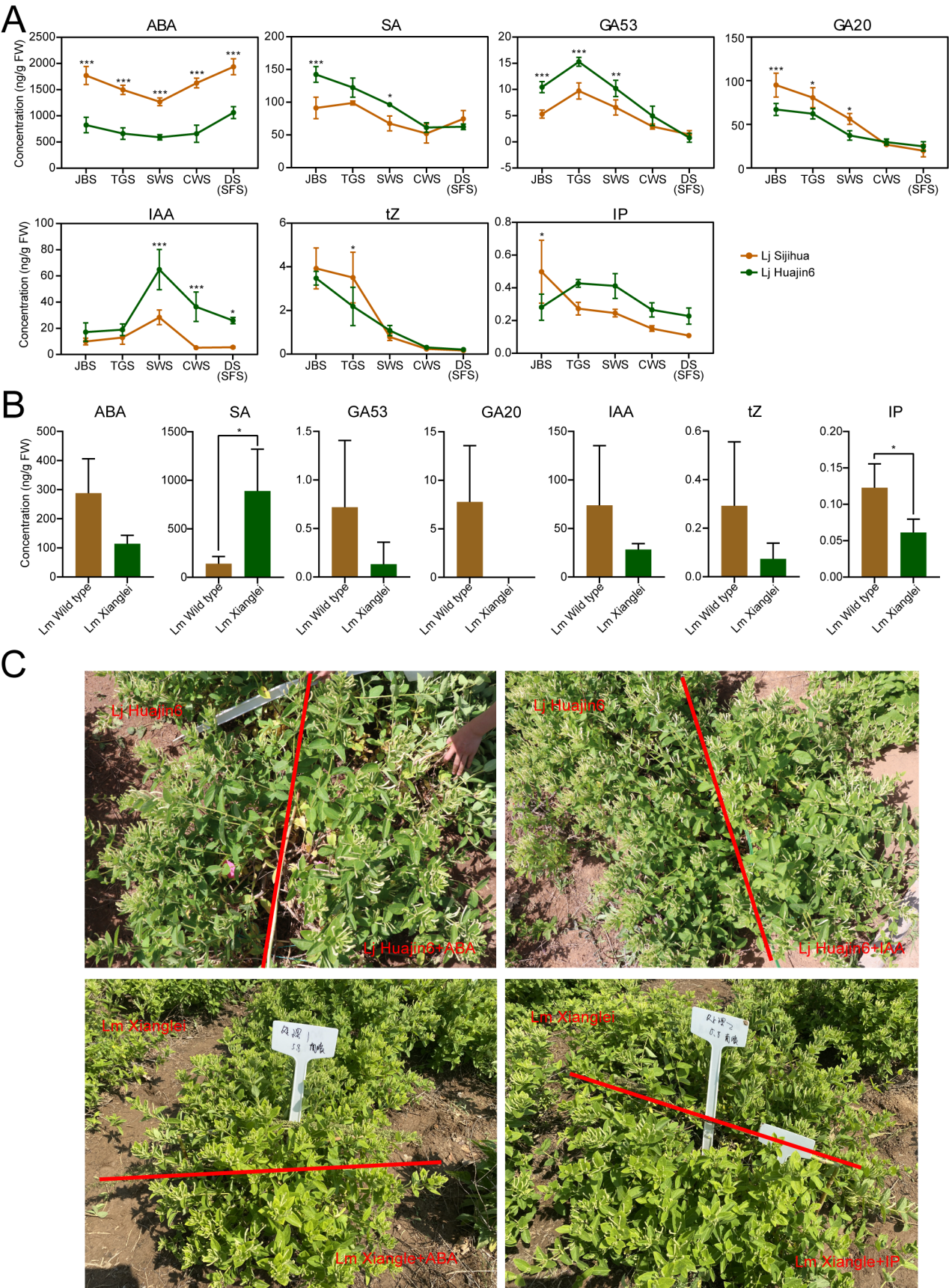
**

**Figure S9**. Plant hormones, including abscisic acid (ABA), salicylic acid (SA), indole acetic acid (IAA), gibberellic acid (GA), and cytokinin (trans-zeatin [tZ], isopentenyl adenine [IP]) do not facilitate flower opening in *L. japonica and L. macranthoides.* (A-B) Concentration of plant hormones in different stages of *L. japonica (A)* and *L. macranthoides* (B) flower buds. Data are expressed as mean ± SD (^*^*P* < 0.05, ^**^*P* < 0.01, ^***^*P* < 0.001, one-way ANOVA, n=3). (C) Phenotypic comparison of *L. japonica* and *L. macranthoides* flowers before and after ABA, IAA or IP treatment.

**
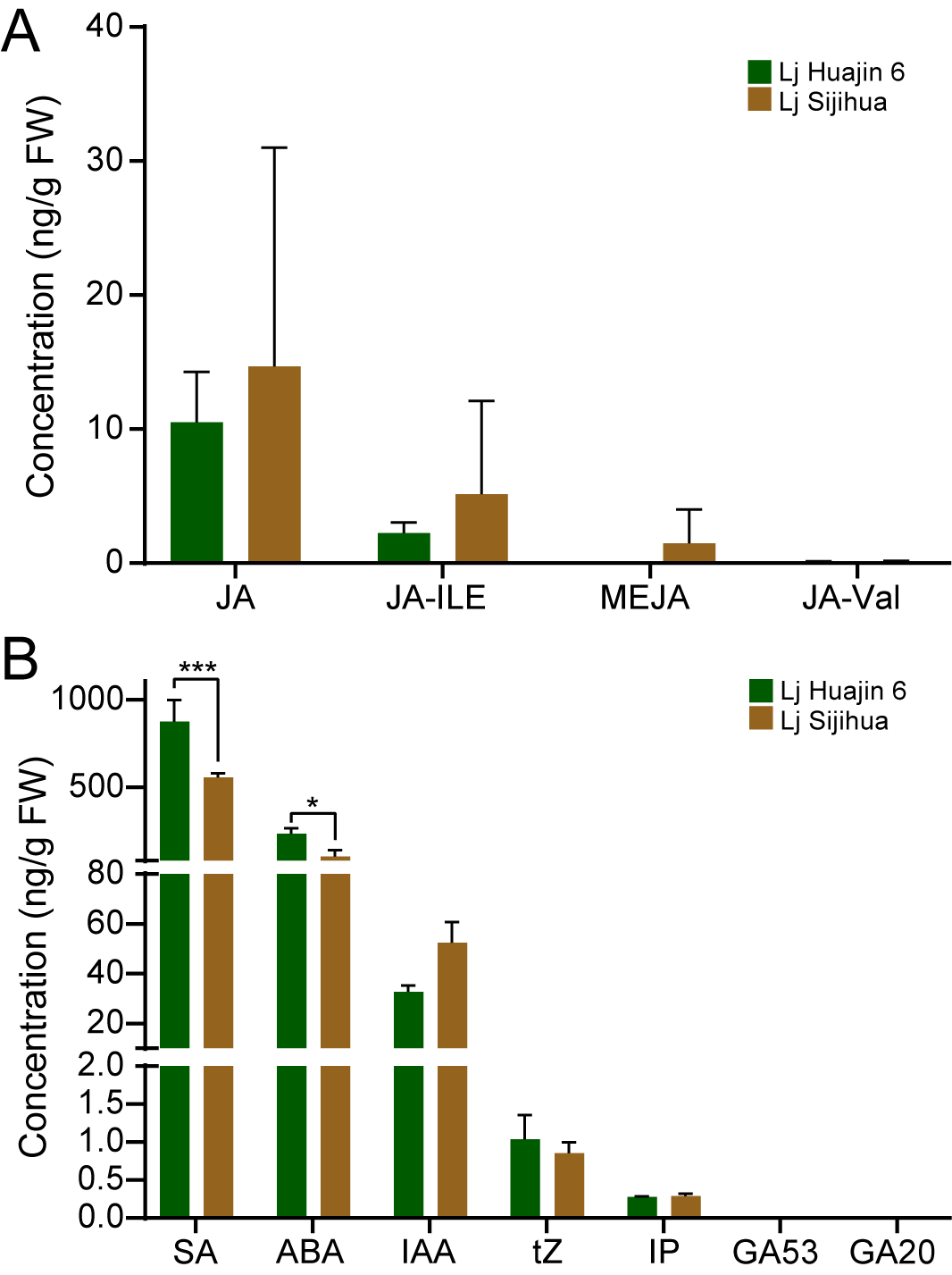
**

**Figure S10**. The concentration of JA has no significant difference in leaves between *Lonicera japonica* ‘Huajin 6’ and *L. japonica* ‘Sijihua’ during the complete white stage. (A) The concentration of jasmonic acid (JA), methyl jasmonate (MeJA), jasmonoyl-isoleucine (JA-Ile), and jasmonoyl valine (JA-Val) in the complete white stage of *L. japonica* ‘Huajin 6’ and *L. japonica* ‘Sijihua’ flower buds. (B) The concentration of other plant hormones, including abscisic acid (ABA), salicylic acid (SA), indole acetic acid (IAA), gibberellic acid (GA), and cytokinin (trans-zeatin [tZ], isopentenyl adenine [IP]) in *Lonicera japonica* ‘Huajin 6’ and *L. japonica* ‘Sijihua’ during complete white stage. Data are expressed as mean ± SD (^*^*P* < 0.05, ^***^*P* < 0.001, one-way ANOVA, n=3).


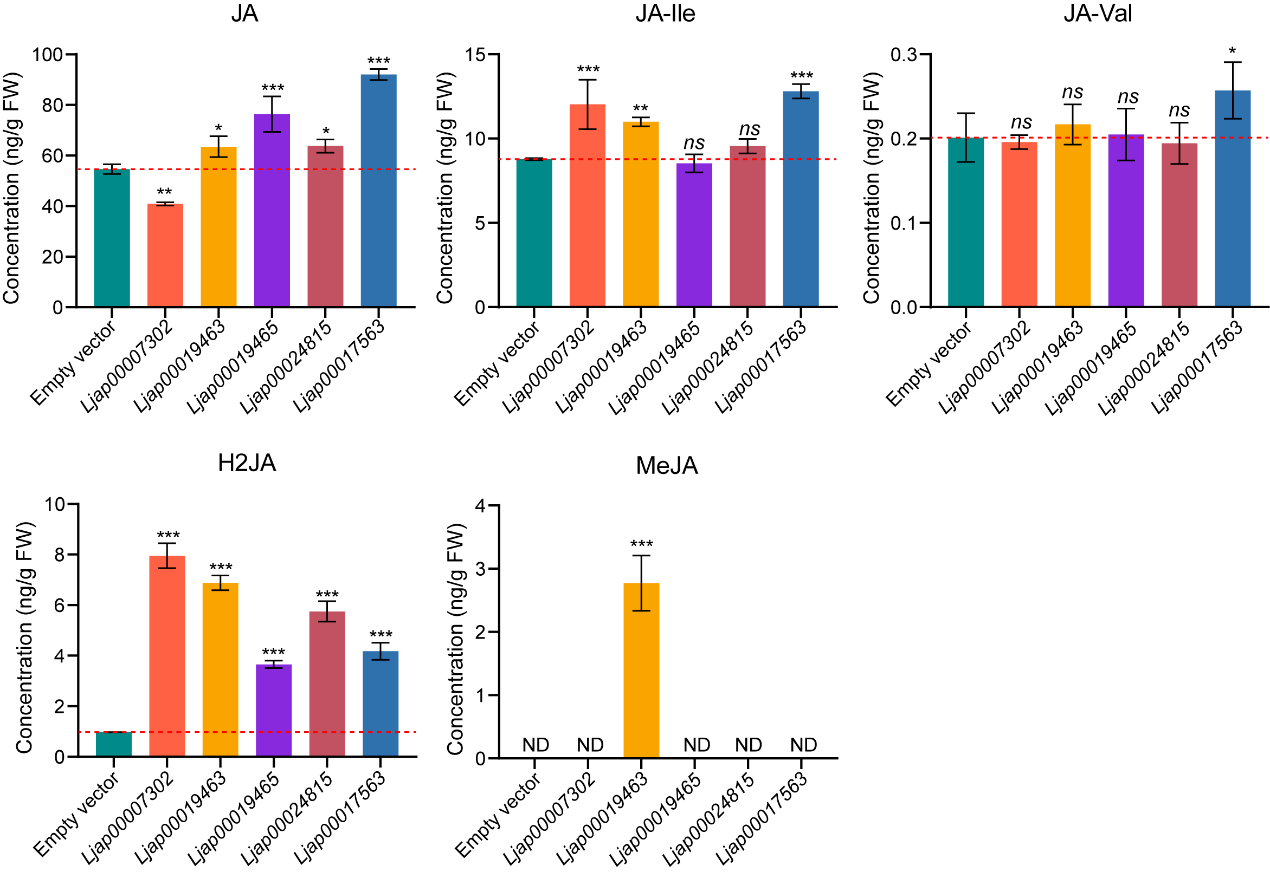


**Figure S11**. Heterologous expression of *Lonicera* *LOX* genes increases jasmonate accumulation in *Arabidopsis*. *Ljap00007302* (*13-LOX* type I), *Ljap00019463* (*13-LOX* type II), *Ljap00019465* (*13-LOX* type II), *Ljap00024815* (*9-LOX*), *Ljap00017563* (*9-LOX*). JA, jasmonic acid; JA-Ile, jasmonoyl-isoleucine; H2JA, dihydrojasmonic acid; MeJA, methyl jasmonate. Data are expressed as mean ± SD (^ns^ *P* > 0.05, ^*^*P* < 0.05, ^**^*P* < 0.01, ^***^*P* < 0.001, one-way ANOVA *vs.* empty vector, n = 3. ND: not detected).
